# Anti-β2GPI/HLA-DR Antibody, Chronic Endometritis, and Uterine Endometrial Microbiome in Women with Recurrent Pregnancy Loss: A Prospective Cohort Study

**DOI:** 10.3390/microorganisms14030544

**Published:** 2026-02-27

**Authors:** Hideto Yamada, Yosuke Ono, Hajime Ota, Yuta Kobayashi, Yoshiyuki Fukushi, Shinichiro Wada, Hisashi Arase

**Affiliations:** 1 Center for Recurrent Pregnancy Loss, Teine Keijinkai Hospital, 1-40, 12-Chome, Maeda, Teine-ku, Sapporo 006-8555, Hokkaido, Japan; 2 Department of Obstetrics and Gynecology, University of Yamanashi, 1110 Shimokato, Chuo 409-3898, Yamanashi, Japan; nadal.babolat@hotmail.co.jp; 3 Department of Obstetrics and Gynecology, Teine Keijinkai Hospital, 1-40, 12-Chome, Maeda, Teine-ku, Sapporo 006-8555, Hokkaido, Japan; hjm.ohta@gmail.com (H.O.); y.koba1021@gmail.com (Y.K.); kohta294@yahoo.co.jp (Y.F.); wa_shin_2002@yahoo.co.jp (S.W.); 4 Laboratory of Immunochemistry, World Premier International Immunology Frontier Research Center, Suita 565-0871, Osaka, Japan; arase@biken.osaka-u.ac.jp; 5 Department of Immunochemistry, Research Institute for Microbial Diseases, Suita 565-0871, Osaka, Japan; 6 Center for Advanced Modalities and DDS, Suita 565-0871, Osaka, Japan; 7 Center for Infectious Disease Education and Research, The University of Osaka, Suita 565-0871, Osaka, Japan

**Keywords:** β2-glycoprotein I, chronic endometritis, endometrial microbiome, HLA-DR, recurrent pregnancy loss

## Abstract

Anti-β2GPI/HLA-DR antibody, chronic endometritis (CE), and endometrial dysbiosis are likely to be associated with the etiologies of recurrent pregnancy loss (RPL). This prospective cohort study aimed to investigate these new risk factors together with conventional causes for RPL, and to evaluate pregnancy outcomes in women individually treated. A total of 87 women with RPL underwent conventional assessment together with anti-β2GPI/HLA-DR antibody measurements, CD138 immunohistochemistry for CE, and 16S rRNA sequence analysis for endometrial microbiome. Women with anti-β2GPI/HLA-DR antibody, CE, and endometrial dysbiosis received low-dose aspirin and heparin, antibiotics, and probiotics, respectively. Pregnancy outcomes of the participants were assessed. Anti-β2GPI/HLA-DR antibody, CE, non-*Lactobacillus*-dominant microbiome (NLDM)-1 (*Lactobacillus* + *Bifidobacterium* < 80%), and NLDM-2 (*Lactobacillus* without *iners* + *Bifidobacterium* < 80%) were detected in 16 (18.4%), 22 (25.3%), 27 (31.0%), and 46 (52.8%) women, respectively. Based on conventional assessment, 65.5% of women with RPL were classified as unexplained etiology; however, the percentage reduced to 16.1% when these new tests were assessed together. All 9 pregnancies with anti-β2GPI/HLA-DR antibody, 13 (92.9%) of 14 pregnancies with CE, and 24 (92.3%) of 26 pregnancies with NLDM-2 resulted in live birth. Assessment of these new tests may be clinically useful for reducing the proportion of unexplained RPL, and for providing high live birth rates if women receive relevant treatments.

## 1. Introduction

Recurrent pregnancy loss (RPL) is defined as the loss of ≥2 pregnancies and affects 0.8–1.4% of couples who desire to have a baby [1,2]. The pathogenesis of RPL involved a variety of causes and risk factors, including abnormal uterine morphology, thyroid dysfunction, antiphospholipid syndrome, thrombophilic disorder, and chromosome abnormality of a couple. However, the etiology of >50% of RPL is unknown, and therefore designated as unexplained RPL [3,4]. Further, the mechanism underlying the pathology of unexplained RPL remains unclear. Some studies have proposed immunological abnormalities, including natural killer cells [5], Th1/Th2 balance [6], and regulatory T cells [7], for the pathophysiology underlying unexplained RPL.

A new concept has emerged, indicating the release of autoantibodies in response to misfolded protein complexes that form with human leukocyte antigen (HLA) class II molecules carrying alleles related to disease susceptibility [8,9]. Among these autoantibodies (so-called neoself antibodies), Tanimura et al. [10]. recognized the involvement of anti-β2GPI/HLA-DR antibodies, which targets the complex formed with β2-glycoprotein I (β2GPI) and HLA-DR, in the pathogenesis of RPL and pregnancy complications associated with antiphospholipid syndrome (APS) [11]. They discovered β2GPI/HLA-DR antigens expressed on the endothelial cells within the decidua of miscarried placentas from women with obstetric APS and suggested that autoantibodies targeting these antigens disrupt placental function.

Conversely, chronic endometritis (CE) is histologically diagnosed as the infiltration of plasma cells into the uterine endometrial stroma, although universal criteria for the CE diagnosis have not been determined [12,13]. Some studies have demonstrated possible adverse effects of CE on human reproduction [14,15,16]. The incidence of CE is reported to be 2.8–56.8% in infertility, 14–67.5% in recurrent implantation failure (RIF), and 9.3–67.6% in RPL, with a wide range [17]. McQeen et al. [18] reported that 56% of women with RPL had CE, and the women without CE showed higher birth rates than those with untreated CE, but with close statistical significance (*p* = 0.08). Immunohistochemistry of the plasma cell marker CD138 (syndecan-1) is a more reliable method than hematoxylin–eosin staining in terms of plasma cell detection, and is used clinically to diagnose CE [17,18]. A high number of CD138-positive cells in the uterine endometrium was a risk for miscarriage in subsequent pregnancies of women with RPL or RIF [19].

Recently, microbiome analyses with 16S ribosomal RNA (rRNA) analysis using a next-generation sequencer have become popular in reproductive techniques [20,21]. Moreno et al. [22] first investigated the uterine endometrium microbiome in infertility using this technique and revealed that implantation, pregnancy, and live birth rates in in vitro fertilization and embryo transfer (IVF-ET) increased in women with *Lactobacillus*-dominant microbiome (LDM) compared with those with non-*Lactobacillus*-dominant microbiome (NLDM). In a prospective cohort study, Shi et al. [23] assessed the vaginal microbiome in women with threatened premature labor using 16S rRNA sequence analysis and revealed that increases in *Ureaplasma* species and decreases in *Lactobacillus* species were associated with subsequent preterm birth. Further, their cohort study evaluated the uterine endometrial microbiome in women with RPL, and they found that increases in *Ureaplasma* and *Gardnerella* species and decreases in *Lactobacillus* species were associated with preterm birth and miscarriage of a fetus with normal chromosome karyotype in their subsequent pregnancies, indicating that the endometrial dysbiosis before pregnancy can cause adverse pregnancy outcomes [24].

However, the guidelines on RPL in Europe [2] or Japan [25] still do not recommend etiological assessment of anti-β2GPI/HLA-DR antibody, CE, or endometrial microbiome. Therefore, the proportion of causes and risk factors for RPL, including anti-β2GPI/HLA-DR antibody, CE, and endometrial dysbiosis, as well as conventional etiologies, remains undetermined. For the first time, this prospective cohort study investigated these new risk factors together with conventional causes/risk factors for RPL. Furthermore, pregnancy outcomes in women who received individual treatments for anti-β2GPI/HLA-DR antibody positivity, CE, and endometrial dysbiosis were assessed in an observational study.

## 2. Materials and Methods

### 2.1. Study Design and Participants

This prospective cohort study was conducted between April 2021 and August 2025 at Teine Keijinkai Hospital, adhering to the principles of the Declaration of Helsinki. The institutional review boards approved the study protocol (Approval #2-020387-00 and #2-020090-02), and written informed consent was obtained from all participants. The study enrolled women diagnosed with RPL who underwent serum anti-β2GPI/HLA-DR antibody measurement, together with an endometrial biopsy for CE assessment with CD138 immunohistochemistry, and endometrial microbiome analysis (16S rRNA sequencing) within the same menstrual cycle. Participants with PRL underwent conventional assessment of the causes and risk factors for PRL, including ultrasound examinations to detect uterine morphological abnormalities, serum tests of thyroid function (i.e., thyroid stimulating hormone and free thyroxine), chromosomal karyotyping of peripheral blood obtained from couples, measurements of serum levels of antiphospholipid antibodies [aPLs, i.e., lupus anticoagulant (LA), immunoglobulin (Ig) G and M anti-cardiolipin antibody (aCL), IgG/M anti-β2-glycoprotein I antibody (aβ2GPI), and β2GPI-dependent anti-cardiolipin antibody (aCL/β2GPI)], and hemostatic molecular markers [i.e., activity levels of protein S (PS), protein C (PC), and coagulation factor XII (FXII)]. Exclusion criteria included women with malignancy, and antibiotic use or probiotics or prebiotics intake within two menstrual cycles before the endometrial biopsy. RPL was defined as a history of ≥2 pregnancy losses before 22 weeks of gestation.

### 2.2. Measurement of Anti-β2GPI/HLA-DR Antibodies

To analyze autoantibody levels specific to the β2GPI/HLA-DR complex and not HLA-DR alone, we modified a previously described method to determine anti-β2GPI/HLA-DR antibody levels [10]. Complementary DNA (cDNA) was prepared from pooled human peripheral blood mononuclear cells (3H Biomedical, Uppsala, Sweden) and cloned into pME18S or pCAGGS expression vectors. 293T cells were transiently transfected using Polyethylenimine Max (Polyscience, Niles, IL, USA). GFP-labeled cells expressing the β2GPI/HLA-DR complex and DsRed labeled cells expressing HLA-DR were generated by transfecting GFP, β2GPI, HLA-DRA*01:01, and DRB1*07:01, or DsRed, HLA-DRA*01:01, and DRB1*07:01 into 293T cells, respectively. A single lot of β2GPI/HLA-DR transfected and HLA-DR transfected cells were aliquoted (3 × 10^6^ cells per tube) with 500 μL of the cryoprotectant medium (90% fetal bovine serum and 10% dimethyl sulfoxide), and stored at −80 °C until use. A serum sample from a woman with RPL in which anti-β2GPI/HLA-DR antibody was detectable after a 10^6^-fold dilution was used as a standard throughout this study. The anti-β2GPI/HLA-DR antibody levels of a standard serum was defined as 1000 anti-β2GPI/HLA-DR antibody units (ABH-U).

The mean fluorescence intensity (MFI) of IgG binding to transfected cells in the sample sera were analyzed with flow cytometry. Specific IgG binding to the β2GPI/HLA-DR complex was calculated by subtracting the MFI of IgG binding to cells transfected with HLA-DR alone from cells transfected with both β2GPI and HLA-DR.

Anti-β2GPI/HLA-DR antibody levels in each serum sample were calculated from the standard curve generated by measuring specific IgG binding to the β2GPI/HLA-DR complex in serially diluted standard sera. All measurements were performed in duplicate, and the mean value was defined as the anti-β2GPI/HLA-DR antibody level of the sample. The cut-off value of 73.3 U was determined by data from 374 healthy controls (300 women and 74 men) as the 99th percentile of the antibody distribution after outlier removal [11]. Serum anti-β2GPI/HLA-DR antibody levels were measured at AOI Biosciences Co., Ltd., Tokyo, Japan. Recommendations for the Management of Recurrent Pregnancy Loss 2025 in Japan [25] designate anti-β2GPI/HLA-DR antibodies as selective tests of risk factor assessment for RPL.

### 2.3. Assessment of Chronic Endometritis and Endometrial Microbiome

Endometrial specimens were obtained through aspiration during the midluteal phase, confirmed by the last menstrual period and transvaginal ultrasound. The vaginal wall and perineum were washed with 0.025 W/V% benzalkonium chloride solution and wiped with a clean cotton swab. A sampling pipette (Pipet CuretTM, CooperSurgical, Inc., Trumbull, CT, USA) was then inserted into the uterine cavity. Specimens in the middle portion of the aspiration tube were immersed in a container kit OMNIgene^®^-VAGINAL for microbiome (DNA Genotek Inc., Ottawa, ON, Canada) that contains DNA/RNA stabilizers, and the remaining tissue was immersed in 8 mL of 10% neutral buffered formalin solution for histopathological assessment, including CD138 immunohistochemistry.

Histopathological analyses for CE were conducted at SAPPORO CLINICAL LABORATORY INC., Sapporo, Japan, using immunohistochemical staining for CD138. CD138-positive cells were defined as plasma cells in the uterine endometrium. CE was diagnosed when the plasma cell count was >5.15/10 mm^2^ based on Liu’s method [26,27]. Although McQeen et al. [18] reported an association between CE and live birth rate in women with RPL, Liu et al. [26] first demonstrated an association between CE and uterine endometrial microbiome in infertile women. Therefore, a cut-off level of plasma cell count > 5.15/10 mm^2^/10 mm^2^ was used for CE assessment in this study.

Microbiome analyses were conducted at a certified commercial laboratory (Varinos Inc., Tokyo, Japan), as previously described [27,28]. The variable region 4 (V4)—the hypervariable region of the 16S rRNA gene—was amplified with polymerase chain reaction (PCR) using DNA extracted from tissue specimens. An amplified PCR sample was identified following the Illumina 16S Metagenomic Sequencing Library Preparation protocol. This cohort study set two categories of NLDM-1 and -2. Endometrial dysbiosis was diagnosed as NLDM-1 when relative dominance rates of *Lactobacillus* plus *Bifidobacterium* species were <80%, and as NLDM-2 when relative dominance rates of *Lactobacillus* species, in which *Lactobacillus iners* was excluded, plus *Bifidobacterium* species were <80%. The relative dominance rates of bacterial vaginosis (BV)-associated bacteria, including *Gardnerella*, *Prevotella*, *Atopobium*, *Dialister*, *Anaerococcus*, *Megasphaera*, and *Streptococcus* species, and the presence of *Ureaplasma* and *Mycoplasma* species in the endometrial microbiome were assessed. Recommendations for the Management of Recurrent Pregnancy Loss 2025 in Japan [25] designate CE and endometrial microbiome as research tests of risk factor assessment for RPL.

### 2.4. Assessment of Causes/Risk Factors for RPL, Treatments and Pregnancy Outcome

The proportion of causes/risk factors identified with conventional assessment, together with new risk factors, including anti-β2GPI/HLA-DR antibody, CE, and endometrial microbiome, was assessed in a cohort of women with RPL.

Women who had conventional causes/risk factors for RPL received common treatments, including uterine surgeries, genetic counseling, medication of antithyroid drugs, levothyroxine, and low-dose aspirin (LDA)/unfractionated heparin (UFH).

Pregnancy course of women who had anti-β2GPI/HLA-DR antibody, CE, and endometrial dysbiosis (NLDM-2) with individual treatments was followed up and assessed. Women with a positive test for anti-β2GPI/HLA-DR antibody received LDA (81 mg/day, orally) from the early follicular phase to 27 weeks of gestation, and UFH (5000 units, twice daily, subcutaneously) after a positive pregnancy test until 34−36 weeks of gestation. Women diagnosed with CE were treated with metronidazole (250 mg, three times a day, orally) or doxycycline (100 mg, twice daily, orally) for 7 days before their pregnancies. Women diagnosed with endometrial dysbiosis (NLDM-2) received probiotics [Lactosupple vaginal tablet daily (EIGHT Oh Trading Company, Tokyo, Japan) and MIYA-BM 3 tablets a day, orally (MIYARISAN Pharmaceutical Co., Ltd., Tokyo, Japan)]. Women with NLDM-2 and the presence of BV-associated bacteria and/or *Ureaplasma/Mycoplasma* received metronidazole (250 mg, three times a day, orally) and/or clarithromycin (200 mg, twice daily, orally) for 7 days. Pregnancy outcomes of participants were monitored until January 2026.

### 2.5. Statistical Analysis

Continuous variables were expressed as medians (minimum–maximum) and compared using the Mann–Whitney U test. Categorical variables were expressed as frequencies (%) and compared using Fisher’s exact test or the Chi-square test. The *p*-values of <0.05 were considered statistically significant.

## 3. Results

### 3.1. Clinical Backgrounds and Etiologies of RPL

**Figure 1:**
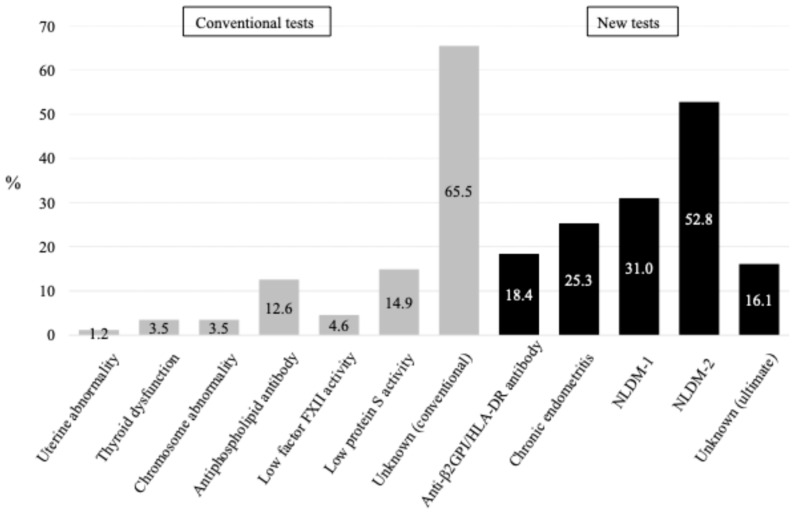
Proportion of causes and risk factors for recurrent pregnancy loss. NLDM; non-*Lactobacillus*-dominant microbiome. NLDM-1; relative dominance rates of *Lactobacillus* plus *Bifidobacterium* species were <80%. NLDM-2; relative dominance rates of *Lactobacillus* species, in which *L. iners* was excluded, plus *Bifidobacterium* species were <80%.

Table 1 shows clinical backgrounds and findings of 87 women with RPL. Uterine morphological abnormality, Hashimoto’s disease, latent hypothyroidism, and balanced reciprocal translocation were found in one (1.2%), two (2.3%), one (1.2%), three (3.5%) women, respectively. Positive aPL tests, low levels of PS, PC, and FXII activity were found in 11 (12.6%), 4 (4.6%), 0, and 13 (14.9%) women, respectively. Excluding women who had any of conventional causes/risk factors for RPL, 56 (65.5%) were diagnosed as having unknown etiology (Figure 1).

The assessment of three new tests for RPL in addition to conventional tests revealed anti-β2GPI/HLA-DR antibody positivity, CE, NLDM-1, and NLDM-2 were detected in 16 (18.4%), 22 (25.3%), 27 (31.0%), and 46 (52.8%) women, respectively (Table 1). In addition to the conventional assessment of causes/risk factors for RPL, if anti-β2GPI/HLA-DR antibody was tested, 47 (54.0%) were classified as having unexplained etiology. Furthermore, if CE, NLDM-1, and NLDM-2 were additionally tested as etiologies for RPL, 36 (41.4%), 21 (24.1%), and 14 (16.1%) were classified as having unexplained etiology, respectively (Figure 1). Of the final 14 women with unexplained RPL, 8 had luteal insufficiency, one had hyperprolactinemia, and the remaining 5 had none of the causes/risk factors.

### 3.2. Differences in Two Categories of NLDM and Endometrial Microbiome

In this study, endometrial dysbiosis was categorized as NLDM-1 and NLDM-2, and the prevalence of BV-associated bacteria and *Ureaplasma*/*Mycoplasma* species are shown in Table 2. The prevalence of BV-associated bacteria in women with NLDM-1 (median 80.6%, range 5.6–100) was higher than that with NLDM-2 but not NLDM-1 (median 0%, 0–14.0, *p* < 0.0001), while the prevalence of *Ureaplasma* or *Mycoplasma* was not significantly different among these categories. Of 27 women with NLDM-1, 25 (92.6%) had >40% of the relative dominance rates of BV-associated bacteria, whereas only one (5.3%) of 19 women with NLDM-2 but not NLDM-1 had the maximum 14.0% of these rates (*p* < 0.01). Women with NLDM-2 but not NLDM-1 had extremely different dysbiosis from those with NLDM-1 in terms of BV-associated bacteria positivity.

### 3.3. Pregnancy Outcome in Women with Anti-β2GPI/HLA-DR Antibody, CE, and Endometrial Dysbiosis

**Figure 2:**
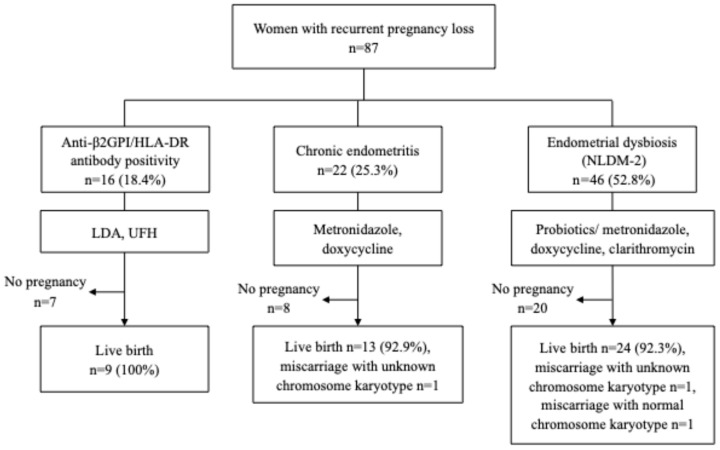
Pregnancy outcomes in women with anti-β2GPI/HLA-DR antibody positivity, chronic endometritis or endometrial dysbiosis. LDA; low-dose aspirin. UFH; unfractionated heparin. NLDM; non-*Lactobacillus*-dominant microbiome. NLDM-2; relative dominance rates of *Lactobacillus* species, in which *L. iners* was excluded, plus *Bifidobacterium* species were <80%. Seven women with anti-β2GPI/HLA-DR antibodies, eight women with CE, and 20 women with NLDM-2 did not become pregnant during the follow-up period, or they were transferred to other clinics.

Of the 16 women with anti-β2GPI/HLA-DR antibodies, 9 became pregnant, and all pregnancies resulted in live birth (100%) until January 2026. Of the 22 women with CE, 14 became pregnant, 13 (92.9%) of whom experienced live birth or ongoing pregnancies beyond 24 weeks of gestation, and one had miscarriage of a fetus with unknown chromosome karyotype. Of the 46 women with NLDM-2, 26 became pregnant, 24 (92.3%) of whom experienced live birth or ongoing pregnancies beyond 24 weeks of gestation, one had miscarriage of a fetus with unknown chromosome karyotype, and one had miscarriage of a fetus with normal chromosome karyotype (Figure 2). Thus, high live birth rates were observed in women who received individual treatments for anti-β2GPI/HLA-DR antibody positivity, CE, or endometrial dysbiosis (NLDM-2). One woman with anti-β2GPI/HLA-DR antibody positivity, CE, and endometrial dysbiosis experienced live birth. Seven women with CE plus endometrial dysbiosis, two with anti-β2GPI/HLA-DR antibody positivity plus CE, and four with anti-β2GPI/HLA-DR antibody positivity plus endometrial dysbiosis experienced live births. One woman with CE plus endometrial dysbiosis experienced miscarriage of a fetus with normal chromosome karyotype, despite receiving standard treatments.

## 4. Discussion

To the best of our knowledge, for the first time, this prospective cohort study on women with RPL demonstrated the proportion of anti-β2GPI/HLA-DR antibody positivity (18.4%), CE (25.3%), and endometrial dysbiosis (NLDM-1, 31.0%; NLDM-2, 52.8%), together with conventional causes/risk factors of RPL, including uterine abnormality (1.2%), thyroid dysfunction (3.5%), chromosome abnormality of couples (3.5%), antiphospholipid syndrome (12.6%), low activity of FXII (4.6%), and PS (14.9%). Based on conventional assessment, 65.5% of women with RPL were classified as unexplained etiology; however, the percentage decreased to 16.1% when three new tests were assessed at the same time in this cohort. Thus, this study clearly demonstrated that the combined assessment of anti-β2GPI/HLA-DR antibody, CE, and endometrial dysbiosis with conventional tests for RPL markedly reduced the proportion of women with unexplained etiology who actually have no relevant therapeutic option.

Several studies have demonstrated that vaginal *Lactobacillus crispatus* is beneficial to pregnancy, while *Lactobacillus iners* is adverse [29,30,31] and more frequently detected in women with CE than in women without CE [27]. A recent cohort study has demonstrated a higher abundance of *Lactobacillus* species, especially *Lactobacillus crispatus,* in vaginal microbiome during early pregnancy is associated with a greater likelihood of pregnancy continuation beyond 38 weeks of gestation [32]. *Lactobacillus iners* is considered a transitional *Lactobacillus* species with low lactic acid-producing capacity and is known to predominate more readily under inflammatory conditions such as bacterial vaginosis [31]. Furthermore, dominance of *Lactobacillus iners* is associated with lower implantation rates and poorer in vitro fertilization outcomes as compared with dominance of *Lactobacillus crispatus* [33].

In order to investigate the microbiological pathophysiology of NLDM in the uterine endometrium, this cohort study set two categories of NLDM-1 and NLDM-2, which excluded *Lactobacillus iners* from the relative dominance rate of *Lactobacillus* species, and revealed that the prevalence (median 80.6%, range 5.6–100) of BV-associated bacteria in women with NLDM-1 was much higher than that of NLDM-2 but not NLDM-1 (median 0%, range 0–14.0). These findings indicate that NLDM-2 includes varied characteristics of endometrial dysbiosis. Therefore, antibiotics treatments for BV-associated bacteria will be necessary in most women with NLDM-1, while probiotic treatments, but not antibiotics, may be appropriate in most women with NLDM-2 but not NLDM-1. Conversely, *Ureaplasma* species, which are known as preterm birth-associated microorganisms, were detected at similar prevalence in endometrial dysbiosis of NLDM-1 (22.2%), NLDM-2 (21.7%), and NLDM-2, but not NLDM (21.1%). Additional antibiotics treatments appropriate for *Ureaplasma* species such as clarithromycin and azithromycin should be considered in women with any NLDM category and *Ureaplasma* positivity.

Recent studies on anti-β2GPI/HLA-DR antibodies have demonstrated that this autoantibody is involved in not only the pathogenesis of RPL, but also fetal growth restriction, hypertensive disorders of pregnancy [34], arterial thrombosis, infertility, endometriosis, and RIF [35]. The antibody positivity in infertile women is associated with lower implantation rates and expression of the β2GPI/HLA-DR antigen complex in endometriotic lesions [35]. Coordinated expression of β2GPI and HLA-DR antigens on epithelial cells of the eutopic endometrium in women with RIF [36] and on vascular endothelial cells of decidual tissues obtained from miscarriages in women with RPL [10] has been demonstrated. Because β2GPI/HLA-DR antigens are thought to be induced by infection and inflammation [8,9], dysbiosis-associated local inflammation and inflammatory cytokines may enhance β2GPI/HLA-DR antigen presentation pathways in the endometrium. The β2GPI/HLA-DR antigen–antibody reaction-associated inflammatory and thrombotic mechanisms may be causally associated with RPL and RIF.

Prospective cohort studies have assessed whether LDA/UFH treatments improve pregnancy outcomes in women with RPL [37] and infertility receiving IVF-ET [36] who tested positive for anti-β2GPI/HLA-DR antibodies. The former study has demonstrated that LDA/UFH treatments in women with RPL and anti-β2GPI/HLA-DR antibody positivity increase live birth rates while reducing complications of preeclampsia and preterm birth before 34 gestational weeks due to placental insufficiency compared with those treated without LDA/UFH [37]. The latter study on women with infertility receiving IVF-ET has demonstrated that anti-β2GPI/HLA-DR antibody positivity is associated with a higher prevalence of RIF and low implantation rates during IVF-ET. In addition, the LDA/UFH treatments during IVF-ET in women with infertility and anti-β2GPI/HLA-DR antibody positivity increase clinical pregnancy and live birth rates and are independently associated with higher clinical pregnancy rates [36]. Thus, LDA/UFH treatments for anti-β2GPI/HLA-DR antibody positivity are likely to be effective in improving pregnancy outcomes in women with RPL and infertility receiving IVF-ET.

Conversely, CE has been associated with endometrial dysbiosis detected by 16S ribosomal RNA sequencing [27,38], and then, this dysbiosis has been associated with RPL [24]. The endometrial dysbiosis is characterized by increased diversity of bacteria species and decreased relative dominance rates in *Lactobacillus* species [39,40]. A high number of CD138-positive cells in the uterine endometrium was a risk for miscarriage in subsequent pregnancies of women with RPL or RIF [19]. A recent systematic review and meta-analysis involving 1038 women with infertility, RIF, and RPL revealed a strong association between CE and RPL, reporting a CE rate of 37.6% in RPL women compared to 16.4% in controls [odds ratio (OR) 3.59]. They also found a significant association between CE and infertility, with 19.46% CE rate in infertile women compared to 7.7% in controls (OR 2.96), but no significant association between CE and RIF, with 6.35% CE rate in women with RIF and 5.8% in controls [41].

Antibiotics treatments were found to reduce CE with an improvement in live birth rates [18,42]. Furthermore, a systematic review and meta-analysis involving 2154 women with reproductive failures, including RIF and RPL, revealed that women with cured CE had higher ongoing pregnancy/live birth rates (OR 1.57) and clinical pregnancy rate (OR 1.56) than those without CE. The analysis also showed a significantly higher ongoing pregnancy/live birth rates (OR 6.82) and clinical pregnancy rate (OR 9.75) in women with cured CE than in those with persistent CE [43].

The present study of pregnancy outcomes demonstrated clearly high live birth rates in women who received individual treatments for anti-β2GPI/HLA-DR antibody positivity (100%), CE (92.9%), or endometrial dysbiosis (92.3%), although it was not a randomized controlled trial (RCT). Therefore, these new tests in addition to conventional assessment for RPL may be clinically useful for reducing the proportion of unexplained RPL and for providing high live birth rates to women with these etiologies if they receive relevant treatments. This study produced useful clinical information for medical practitioners who specialize in RPL.

This study had several limitations. The number of participants was relatively small, and validation of the findings in a larger cohort is warranted. As this study was observational in nature, causality between anti-β2GPI/HLA-DR antibodies, CE, or endometrial dysbiosis and RPL cannot be established, and potential interactions and bidirectionality should be further investigated. In addition, anti-β2GPI/HLA-DR antibodies were evaluated based on a single measurement, and persistence of positivity was not confirmed by repeat testing at ≥12 weeks. Treatment efficacy was not evaluated using RCT. The proportion of NLDM-2 was found to be more than a half, and treatments with probiotics and/or antibiotics might be not necessary for all women with RPL and NLDM-2. To confirm the findings of this study, large-scale cohort studies and RCTs on therapeutic options are warranted in future.

## 5. Conclusions

This cohort study, for the first time, demonstrated the proportion of anti-β2GPI/HLA-DR antibody positivity, CE, and endometrial dysbiosis together with conventional causes/risk factors of RPL. High live birth rates were observed in women who tested positive for these new tests and received individual treatments. Therefore, these new tests in addition to conventional assessment for RPL may be clinically useful for reducing the proportion of unexplained RPL and for providing high live birth rates to women with these etiologies if they receive relevant treatments.

## Figures and Tables

**Table 1:** Clinical backgrounds and findings of 87 women with recurrent pregnancy loss.

Clinical Backgrounds and Findings	
Age, year	35 (23–45)
Body mass index, kg/m^2^	21.4 (16.2–37.2)
Gravida	3 (2–13)
The number of previous miscarriages	2 (2–11)
Serum levels of anti-β2GPI/HLA-DR antibody, U	26.1 (0–1285.9)
Anti-β2GPI/HLA-DR antibody positivity (≥73.3 U)	16 (18.4%)
Plasma cell count/10 mm^2^ with CD138 staining in the uterine endometrium	1.44 (0–252.6)
Chronic endometritis (plasma cell count > 5.15/10 mm^2^, Liu’s method)	22 (25.3%)
Relative dominance rate of *Lactobacillus* plus *Bifidobacterium* species <80% in endometrial microbiome (NLDM-1)	27 (31.0%)
Relative dominance rate of *Lactobacillus* excluding *L. iners* plus *Bifidobacterium* species <80% in endometrial microbiome (NLDM-2)	46 (52.8%)
**Endometrial Microbiome**	
Number of bacterial species	4.0 (1–17)
Relative dominance rate of *Lactobacilus* species, %	98.8 (0–100)
Relative dominance rate of *Lactobacillus* species excluding *L. iners*, %	40.4 (0–100)
Relative dominance rate *Bifidobacterium* species, %	11.8 (0.1–99.9)
Presence of *Lactobacilus crispatus*	42 (48.3%)
Presence of *Lactobacilus gasseri*	20 (22.9%)
Presence of *Lactobacilus jenseni*	2 (17.2%)
Presence of *Lactobacilus iners*	36 (41.4%)
Relative dominance rate of *Lactobacilus crispatus*, *%*	0 (0–100)
Relative dominance rate of *Lactobacilus gasseri*, %	0 (0–86.7)
Relative dominance rate of *Lactobacilus jenseni*, *%*	0 (0–98.3)
Relative dominance rate of *Lactobacilus iners*, *%*	0 (0–99.9)
Presence of *Bifidobacterium* species	10 (11.5%)
Presence of *Gardnerella* species	29 (33.3%)
Presence of *Prevotella* species	28 (32.2%)
Presence of *Atopobium* species	20 (22.9%)
Presence of *Dialister* species	19 (21.8%)
Presence of *Anaerococcus* species	7 (8.0%)
Presence of *Megasphaera* species	5 (5.7%)
Presence of *Streptococcus* species	12 (13.8%)
Presence of *Ureaplasma* species	16 (18.4%)
Presence of *Mycoplasma* species	2 (2.3%)

Data are shown as median (range) or number (%). NLDM, non-*Lactobacillus*-dominant microbiome.

**Table 2:** Categories of endometrial dysbiosis (non-Lactobacillus-dominant microbiome).

Non-*Lactobacillus*-Dominant Microbiome (NLDM)	Number of Women	A Total of Relative Dominance Rates of *Gardnerella*, *Prevotella*, *Atopobium*, *Dialister*, *Anaerococcus*, *Megasphaera*, and *Streptococcus* Species	Number of Women with *Ureaplasma*/*Mycoplasma*
Relative dominance rates of *Lactobacillus* plus *Bifidobacterium* species <80% in endometrial microbiome (NLDM-1)	27	80.6% (5.6–100) *	6/2
Relative dominance rates of *Lactobacillus* excluding *L. iners* plus *Bifidobacterium* species <80% in endometrial microbiome (NLDM-2)	46	52.8% (0−100)	10/2
NLDM-2 but not NLDM-1	19	0% (0–14.0) *	4/0

Median (range). * *p* < 0.0001 (NLDM-1 vs. NLDM-2 but not NLDM-1).

## Data Availability

The original contributions presented in this study are included in the article. Further inquiries can be directed to the corresponding author.

## Abbreviations

RPL	recurrent pregnancy loss
β2GPI	β2-glycoprotein I
HLA	human leukocyte antigen
APS	antiphospholipid syndrome
CE	chronic endometritis
RIF	recurrent implantation failure
rRNA	ribosomal RNA
IVF-ET	in vitro fertilization and embryo transfer
NLDM	non-*Lactobacillus*-dominant microbiome
PS	protein S
PC	protein C
FXII	coagulation factor XII
PCR	polymerase chain reaction
BV	bacterial vaginosis
LDA	low-dose aspirin
UFH	unfractionated heparin
RCT	randomized controlled trial
